# Epidemiology of Tuberculosis in an Urban Slum of Dhaka City, Bangladesh

**DOI:** 10.1371/journal.pone.0077721

**Published:** 2013-10-21

**Authors:** Sayera Banu, Md. Toufiq Rahman, Mohammad Khaja Mafij Uddin, Razia Khatun, Tahmeed Ahmed, Md. Mojibur Rahman, Md. Ashaque Husain, Frank van Leth

**Affiliations:** 1 Centre for Communicable Diseases, International Centre for Diarrhoeal Disease Research, Bangladesh, Dhaka, Bangladesh; 2 National Tuberculosis Control Program, Directorate General of Health Services, Mohakhali, Dhaka, Bangladesh; 3 Department of Global Health, Academic Medical Centre, University of Amsterdam, Amsterdam Institute for Global Health and Development, Amsterdam, The Netherlands; University of Cape Town, South Africa

## Abstract

**Background:**

The objectives of this study were to assess the tuberculosis (TB) burden and to provide an insight into the type of circulating *M. tuberculosis* species in urban slums of Bangladesh. We also aimed to test the feasibility of a larger transmission study in this setting.

**Methods:**

This cross-sectional study was conducted in an urban slum of Dhaka city. The household members were actively screened to assess the presence of TB-related signs and symptoms; cough ≥3 weeks and body mass index (BMI) <17 kg/m^2^. Sputum specimens from suspects were collected for acid fast bacilli (AFB) microscopy, culture and drug susceptibility testing. Genotyping of *M. tuberculosis* was done using spoligotyping and variable number tandem repeats of mycobacterial interspersed repetitive units typing.

**Results:**

Among 9,877 adult screened for pulmonary TB (PTB), 25 were positive for AFB on microscopy and/or culture and the prevalence of new PTB cases was estimated to be 253/100,000. Only one child TB case was diagnosed among 5,147 child screened. Out of 26 cases, 21(81%) had cough for several duration and 5(19%) did not present with cough at the time of screening. One multidrug resistant case was found. Fifty two percent of all TB cases had BMI <17 kg/m2 (p = <0.001). Among the 20 analyzed isolates, 13 different spoligotype patterns were identified in which 5 clusters contained 12 strains and 8 strains had unique pattern.

**Conclusions:**

The study revealed high prevalence of TB in urban slums. Screening using low BMI can be beneficial among risk group population. It is important to conduct larger study to validate clinical variables like cough <3 weeks and low BMI to define TB suspect and also to investigate the transmission of TB in slum settings.

## Introduction

The global burden of tuberculosis (TB) in 2010 is estimated to be 8.8 million in the form of incident cases, 12 million in the form of prevalent cases [1]. The incidence is estimated indirectly through notification of passively detected patients. The real burden of TB could be markedly higher if active case finding would be employed on larger scale than is now often done.

In many countries, differences in TB incidence between urban and rural areas have been described [2]. TB in urban areas often results specifically among certain urban risk groups, such as slum dwellers who are exposed to poor environmental conditions (overcrowding, poor living conditions) [3]. TB is still believed to be a disease that disproportionately affects the poor and marginalized [4].

The National Tuberculosis Control Programme (NTP) of Bangladesh first adopted the directly observed treatment short course (DOTS) strategy in 1993. The programme rapidly expanded in the following years to almost all areas of the country reaching 100% coverage in 2006 [5]. There are still some gaps in the DOTS services provided for the urban slum dwellers. DOTS strategy is entirely based on passive case finding which is often influenced to a great extent by the treatment seeking behavior of the patients suffering from active TB, social stigmatization, access to health service and even diagnostic delay at health facility [6]. This in turn results in decreased TB case detection with underestimated number of actual TB cases prevailing in the community. Bangladesh had comparatively higher percentage (81%) of notified cases of pulmonary TB (PTB) that were sputum smear-positive (SS+) among the 22 high burden countries with TB [1]. Delay in the diagnosis of these open TB cases can results in transmission of TB among the contacts of the active TB cases and more likely to fuel its transmission in the community apart from increased morbidity and mortality [7].

The prevalence of SS+ TB was found to be higher in the rural population (86.0/100 000) compared to urban (51.1/100 000) in the recently completed (2007–2009) nationwide TB prevalence survey of Bangladesh [8]. A lesser number of cases in urban areas was also notified in the NTP in 2010 [9]. In 2010, the NTP notified a total of 158,709 of all forms of TB cases (103/100 000 population) nationwide, of these 13% were reported from urban areas. However, there are still some pockets in urban area where TB notification rate believed to be higher. One of these is high burden settings like urban slums.

There are still major gaps in our epidemiological knowledge regarding the transmission dynamic of TB despite the fact that TB is endemic and highly prevalent in Bangladesh. Results from one of our previous studies performed in a rural community of Matlab showed that TB in rural Bangladesh is caused primarily by reactivation of latent infections, with the recent emergence of Beijing strain clusters [10]. Urban areas in Bangladesh are densely populated and about one third of the populations are slum dwellers, creating conditions where a high transmission can occur.

Typing of *M. tuberculosis* strains is of potential importance for the epidemiological studies of TB. The most frequently used genotyping methods for *M. tuberculosis* are restriction fragment-length polymorphism (RFLP), which targets the insertion sequence (IS) 6110 transposable element, and spoligotyping [11]. An alternative polymerase chain reaction (PCR)-based technique targets 12 loci containing variable number tandem repeats (VNTRs) of genetic elements named mycobacterial interspersed repetitive units (MIRUs) [12], [13]. This method has a discriminatory power close to that of IS6110 RFLP analysis [14], [15]. These techniques are well implemented both in urban and rural settings, in several studies related to transmission dynamics of TB across the globe [10], [16].

The aims of this study were to investigate the burden of active TB in an urban slum of Dhaka city with increased number of case detection based on active symptom screening and also to provide an insight into the type of circulating *M. tuberculosis* species. We also aimed to test the feasibility of a larger study to investigate TB transmission in this setting.

## Methods

### Study setting

**Figure 1:**
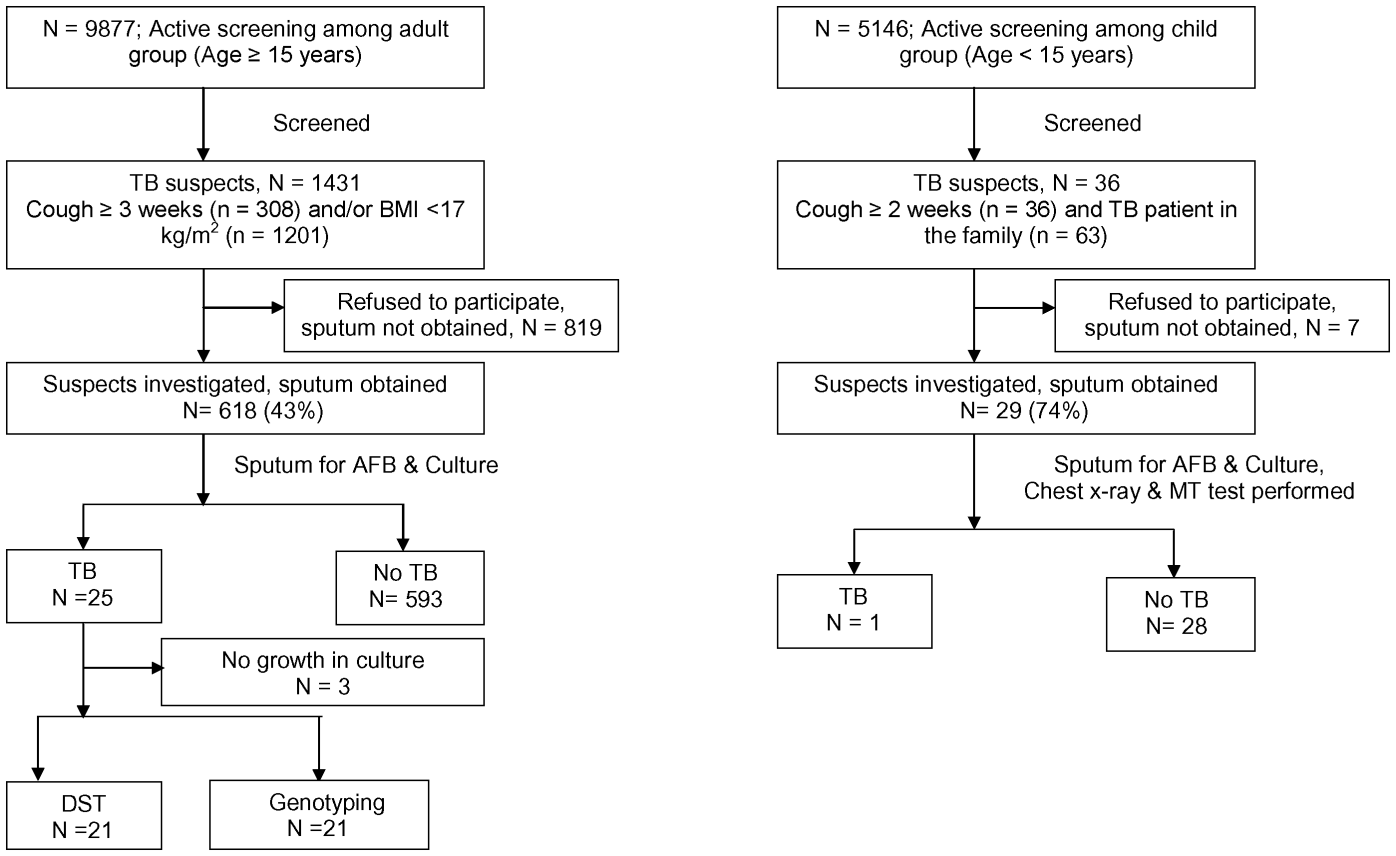
Map of the section-11, Mirpur area showing the location of unique and clustered strains isolated from tuberculosis cases.

The study was conducted at a densely populated low income urban slum (Mirpur slum) in Dhaka, Bangladesh (Figure 1). Mirpur is one of the 41 thanas of Dhaka City with a population of about one million in an area of 59 square kilometers. Mirpur Thana is divided into 14 sections and we conducted the activities of this project in section 11. The population of section 11 is approximately 50,000. There are several camps in Section 11 and these are inhabited by poor and lower middle class families, residential and sanitary conditions are typical of any congested urban settlement.

### Ethics and consent

The study protocol was reviewed and approved by the Research Review Committee and the Ethical Review Committee of International Centre for Diarrhoeal Disease Research, Bangladesh (icddr,b). The participants were enrolled into the study only after receiving informed written consent from themselves or from their parents/guardians if they were children.

### Study procedure

This cross-sectional house-to-house survey was conducted from August 2009 to December 2010. Individuals not present at the household during the first house visit were attempted to be included on at least one subsequent visit. The team consisting of two trained field assistants (FAs) and one research physician visited the study area on a daily basis and screened them for TB symptoms using simple standardized questionnaires. The socio-demographic characteristics, history regarding TB symptoms and other relevant information were collected from the consenting participants. Individuals having productive cough for three or more weeks with or without other clinical presentation were identified as suspects for PTB. We also considered a body mass index (BMI) <17 kg/m^2^ as a single inclusion criteria for the study as one of our previous study showed more likelihood of developing TB among individuals with BMI<17 [17]. In case of children, we considered a child to be a TB suspect if he/she had cough ≥2 weeks and presence of TB patient in the family [18]. Three sputum specimens from each PTB suspect were collected by the FAs after further clinical evaluation of the suspects by the research physician. The first sputum specimen was obtained immediately after identifying the subject as suspect. The second specimen was overnight sputum collected on the next morning and the third was spot during collection of the second sputum specimen. PTB suspects who did not provide consent to provide sputum specimens in the study were re-invited at least once. The specimens were immediately brought to icddr,b Tuberculosis Laboratory in a cool box for acid-fast bacilli (AFB) microscopy, culture and drug susceptibility testing (DST). We also arranged chest X-ray and Mantoux test for child TB suspects in an adjacent private diagnostic facility. Modified Kenneth-Jones' criteria was used to diagnose child TB [19], [20].

### Laboratory investigations

Concentrated sputum smears were examined for AFB using the Ziehl-Neelsen staining under light microscope. Sputum specimens were decontaminated following the Petroffs' NaOH method [21]. Conventional culture using Löwenstein-Jension solid media were used for TB culture using the method as described previously [22].

The standard proportion method was followed for DST of *M. tuberculosis* isolates to isoniazid (0.2 mg/l), rifampicin (40 mg/l), ethambutol (2 mg/l) and streptomycin (4 mg/l) [23]. An isolate was considered resistant to a given drug when any growth of 1% or more above the control was observed in each drug- containing quadrant plate.

Spoligotyping was performed as previously described by Kamerbeek et al. [24] with minor modifications. The direct repeat (DR) region was amplified by PCR with oligonucleotide primers derived from the DR sequence. Mycobacterial genomic DNA was extracted from cultured cells as described previously [25], [26]. The amplified product was hybridized to a set of 43 immobilized oligonucleotides, each corresponding to one of the unique spacer DNA sequences within the DR locus. Detection of hybridizing DNA was by the chemiluminescent ECL method (Amersham) [26], [27] and by exposure to X-ray film (Hyperfilm ECL: Amersham) in accordance with the instructions of the manufacturer.

PCR and calculation of MIRU copy number per locus were carried out as described previously [13], [28], [29]. All isolates were typed using 11 loci (2, 4, 10, 16, 23, 24, 26, 27, 31, 39 and 40) VNTR-MIRU typing. Most of the strains failed to give a PCR product with primers for the MIRU 20 locus, as seen in our previous study [29]; therefore, the results of MIRU 20 locus were excluded from this study.

### Case definitions for TB disease

The diagnosis of TB was made according to the case definition given by the NTP depending on the site and bacteriological status [30]. SS+ PTB was defined as a positive sputum smear confirmed with a second positive smear or culture of *M. tuberculosis* or chest radiological X-ray abnormalities consistent with active TB; and smear negative PTB was defined by two positive cultures of *M. tuberculosis* while three sputum specimens negative for AFB [30].

### Epidemiological investigation

A cluster was defined as two or more isolates from different patients with identical spoligotype or MIRU patterns, whereas nonclustered patterns were referred to as unique [10]. Clustered patients were investigated to further establish or strengthen potential epidemiological connections in place, time, and person among cluster members. Participants were being considered to share a strong epidemiological link if they would have had been in the same workplace, household, village or area at overlapping times (even a known single exposure to patients).

### Statistical analysis

Data were entered and analyzed using the Statistical Package for Social Sciences (SPSS) version 17.0. Univariate analyses were performed to examine the association between demographic and clinical variables of TB cases. *P*<0.05 was considered as evidence of significant difference. To identify the independent risk factors for TB and non TB, adjusted odds ratio (AOR) and 95% CI were calculated by logistic regression analysis.

## Results

**Figure 2:**
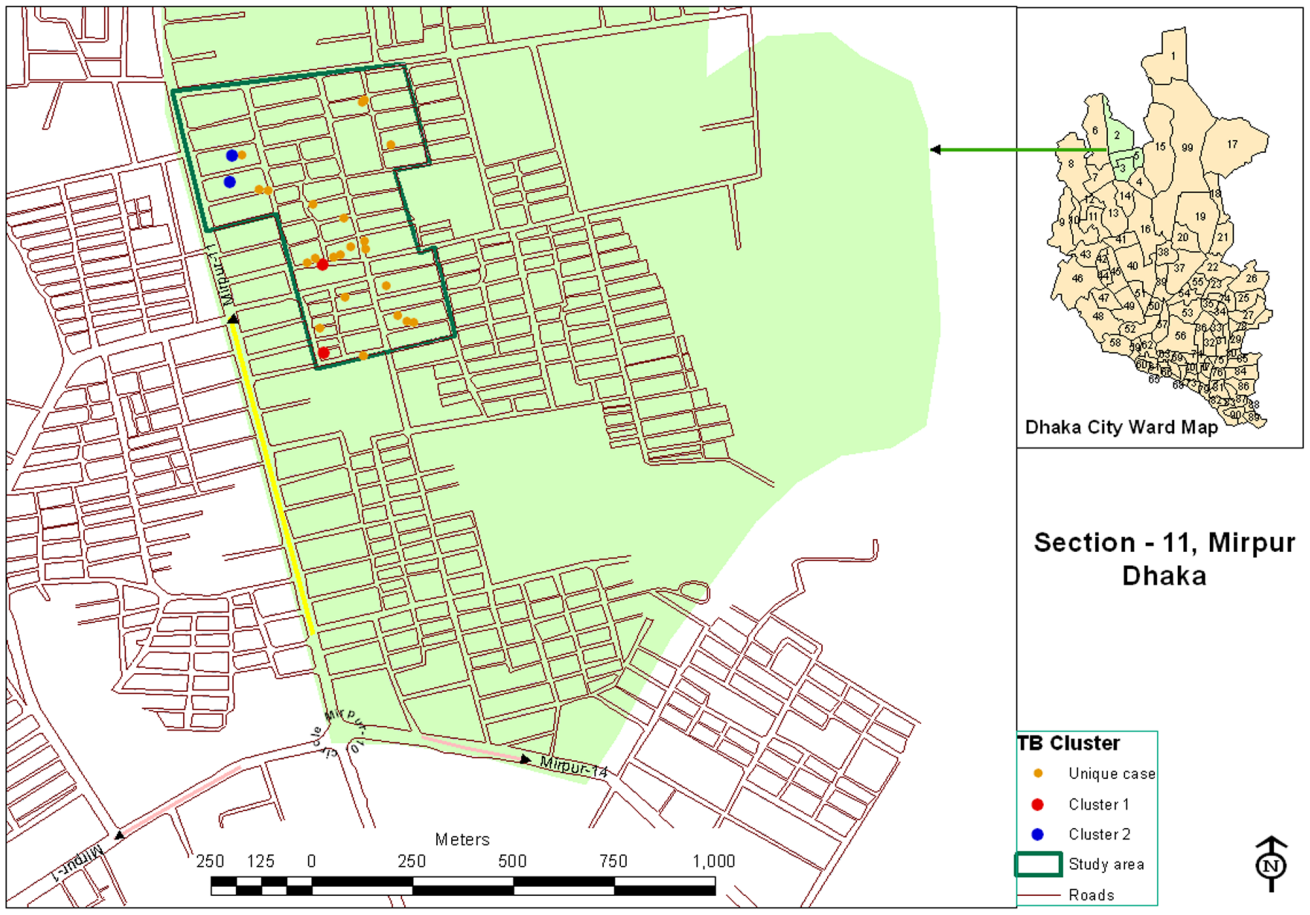
Screening profile of individuals suspected of having tuberculosis.

A total of 3716 households were visited and 16,706 eligible participants belonged to those households; of them 15,024 (90%) consented subjects were screened during 17 month period. Of these 9,877 (66%) were aged 15 years or more (adults) and 5,147 (34%) were aged below 15 years (children) (Table 1). Out of 1431(14% of 9,877) adult PTB suspects, sputum specimens were collected from 618 (43%) suspects. Majority (87%) of these suspects who were unable to provide sputum specimen were initially identified as a suspect because of their low BMI even in the absence of cough for any duration. Among the child population, 36 child suspects were identified and sputum smear microscopy and relevant tests were performed in 29 child suspects (Figure 2). We have detected 26 (4% of 647 suspects) TB cases during this time span. Out of 26 cases, 16 (62%) were male and 10 (38%) were female, with a ratio of 1.6∶1. Only one child TB case was diagnosed. Out of the 25 adult TB cases identified in our study, 19 (76%) were smear positive; and the remaining 6 (24%) cases were smear negative but showed growth in culture. The estimated number of new pulmonary TB cases (AFB and/or culture) was 253/100,000 population and the estimated number of new SS+ TB cases was 192/100,000 populations; among the subjects aged ≥15 years, who participated in the study. All 26 identified PTB cases were brought under treatment by DOTS programme.

**Table 1:** Characteristics of overall study population.

Variables		Study population			
		All screened (n = 15024)	TB (n = 26)	Non-TB (n = 14998)	
Category	Sub categories	number (%)	number (%)	number (%)	p Value
**Demographics**					
**Sex**	Male	6757 (45.0)	16 (61.5)	6741 (44.9)	0.09
	Female	8267 (55.0)	10 (38.5)	8257 (55.1)	
**Age**	0–14 yrs	5151 (34.3)	1 (3.8)	5150 (34.3)	**<0.001**
	15–24 yrs	3739 (24.9)	7 (26.9)	3732 (24.9)	
	25–34 yrs	2601 (17.3)	4 (15.4)	2597 (17.3)	
	35–44 yrs	1665 (11.1)	4 (15.4)	1661 (11.1)	
	45–54 yrs	926 (6.2)	6 (23.1)	920 (6.1)	
	55–64 yrs	553 (3.7)	2 (7.7)	551 (3.7)	
	65+ yrs	389 (2.6)	2 (7.7)	387 (2.6)	
**Occupation** *	Self-employed	1681(17.0)	10 (40.0)	1671 (17.0)	**0.02**
	Business	481(4.9)	2 (8.0)	479 (4.9)	
	Service	2490 (25.2)	5 (20.0)	2485 (25.2)	
	Unemployed	1982 (20.1)	5 (20.0)	1977 (20.1)	
	Housewife	3236 (32.8)	3 (12.0)	3233 (32.8)	
**Smoking**	No	14346 (95.5)	19 (73.1)	14327 (95.5)	<0.001
	Yes	678 (4.5)	7 (26.9)	671 (4.5)	
**Symptoms**					
**Cough**	No	14488 (96.4)	5 (19.2)	14483 (96.6)	<0.001
	Yes	537 (3.6)	21 (80.8)	516 (3.4)	
	<2 weeks	157 (29.2)	1(4.8)	156 (30.2)	**0.02**
	≥2 weeks <3	47 (8.8)	3 (14.3)	44 (8.5)	
	≥3 weeks	333 (62.0)	17 (81.0)	316 (61.2)	
**Haemoptysis**	No	14989 (99.8)	24 (92.3)	14965 (99.8)	<0.001
	Yes	35 (0.2)	2 (7.7)	33 (0.2)	
**Evening rise of temperature**	No	14898 (99.2)	14 (53.8)	14884 (99.2)	<0.001
	Yes	126 (0.8)	12 (46.2)	114 (0.8)	
**Chest pain**	No	14847 (98.8)	19 (73.1)	14828 (98.9)	<0.001
	Yes	177 (1.2)	7 (26.9)	170 (1.1)	
**Shortness of breathe**	No	14884 (99.1)	11 (42.3)	14873 (99.2)	<0.001
	Yes	140 (0.9)	15 (57.7)	125 (0.8)	
**Risk factors**					
**Previously diagnosed as TB**	No	14784 (98.4)	17 (65.4)	14767 (98.5)	<0.001
	Yes	240 (1.6)	9 (34.6)	231(1.5)	
**Exposure to TB patient**	No	13991 (93.1)	25 (96.2)	13966 (93.1)	**>0.1**
	Yes	1033 (6.9)	01 (3.8)	1032 (6.9)	
**BMI** *	BMI (≥17.0)	8676 (87.8)	12 (48.0)	8664 (87.9)	**<0.001**
	BMI (<17.0)	1201 (12.2)	13 (52.0)	1188 (12.1)	

All value are n (%). p values are comparing TB patients (n = 26) against no. no-TB (n = 14998).

* Occupation and BMI were calculated among the adult group only;

Fisher exact test are shown in boldface font.

A number of clinical variables were assessed for an association with TB in this study. Out of 26 diagnosed cases; 21(81%) had cough and 5(19%) did not present with any cough at the time of active screening, they were suspected on the basis of their poor nutritional status (Table 1). Malnutrition, as defined by low BMI (<17 kg/m^2^), was also associated with TB; this can either be a risk factor or result of TB. Fifty two percent of all TB cases had BMI <17 kg/m^2^ (p = <0.001) (Table 1). Thirty five percent of the identified TB cases had a previous history of TB (p = <0.001). Other clinical variables which were associated with TB were fever, haemoptysis, chest pain and shortness of breath (not statistically significant). No significant association was observed between TB and diabetes mellitus, exposure to TB patients, and alcohol consumption. After adjusting for confounding factors, a risk factor analysis showed that a higher likelihood of developing active TB was associated with smoking, previous history of anti-TB treatment and low BMI (Table 2).

**Table 2:** Odds ratios (ORs) for TB by socio demographic characteristics and potential risk factors.

Variables		Unadjusted		Adjusted	
Category	Sub categories	OR (95% CI)	*P* Value	AOR (95% CI)	*P* Value
**Socio demographic**					
Sex	Female	1.00			
	Male	1.960 (0.9 – 4.3)	0.09		
Age	0–14 yrs	1.00			
	15–24 yrs	9.7 (1.2–78.5)	0.03		
	25–34 yrs	7.9 (0.9 –71.0)	0.06		
	35–44 yrs	12.4 (1.4–111.0)	0.02		
	45–54 yrs	33.6 (4.0–279.3)	0.001		
	55–64 yrs	18.7 (1.7–206.5)	0.01		
	65+ yrs	26.6 (2.4–294.2)	0.007		
Occupation*	Self-employed	1.0			
	Business	6.4 (1.8 – 23.4)	0.005		
	Service	4.5 (0.8 – 26.9)	0.10		
	Unemployed	2.2 (0.5 – 9.1)	0.29		
	Housewife	2.7 (0.7 – 11.4)	0.17		
Smoking	No	1.0		1.0	
	Yes	7.9 (3.3 – 18.8)	<0.001	3.7 (1.5– 9.3)	0.005
**Risk factors**					
Previously diagnosed as TB	No	1.0		1.0	
	Yes	33.8 (14.9 –76.7)	<0.001	15.7 (6.6–37.3)	<0.001
BMI*	BMI (≥17.0)	1.0		1.0	
	BMI (<17.0)	7.9 (3.6–17.4)	<0.001	5.3 (2.3–12.0)	<0.001

OR =  odds ratio; AOR =  adjusted odds ratio; CI =  confidence interval; adjusted odds ratio are not presented for variables with *P* values more than 0.1; * Occupation and BMI were calculated among the adult group only.

Out of 26 cases, DST was done on 20 strains of *M. tuberculosis* available. Five cases were not available (one was unable to produce valid culture result, two started anti-TB treatment before collection of specimens for culture, one specimen was missing for culture and the child TB case was negative on culture) for DST and another one was excluded from the analysis as it was non tuberculous mycobacterial (NTM) strain. One (5%) strain was resistant to streptomycin alone and 1 (5%) was resistant to all four drugs. The remaining 18 (90%) strains were susceptible to first line drugs. The DR-TB case was notified to the NTP for subsequent management.

Twenty isolates from *M. tuberculosis* cultures were spoligotyped. This analysis revealed 13 different patterns, 12 of which were grouped into 5 clusters and remaining 8 isolates were unique. About 60% of the isolates were clustered by spoligotyping (Table 3). Twenty five percent of the isolates were of East African Indian (EAI) type [31], which corresponds to the ancestral TbD1+ *M. tuberculosis* type [32], also named Indo-Oceanic cluster [33]. There was a single cluster, EAI5 among the EAI type strains. The other strains of the family were EAI6_BGD1 and EAI-1-SOM types. EAI5 was also the most predominant of the EAI type consisting of 3 isolates. Other clusters included the Beijing family and T-family strains consisted of two isolates in each cluster (Table 3). Using MIRU typing, only 4/20 (20%) strains could be grouped into two clusters. Remaining sixteen isolates (80%) exhibited unique (non-clustered) patterns.

**Table 3:** Clusters and unique *M. tuberculosis* strains determined by various typing methods and distribution of spoligotyping-defined phylogenetic clades.

	Strain	Spoligo pattern	Phylogenetic clade	VNTR-MIRU type Allele profile
1	MP132901	□□□□□□□□□□□□□□□□□□□□□□□□□□□□□□□□□□▪▪▪▪▪▪▪▪▪	Beijing	22435173512
2	MP348502	□□□□□□□□□□□□□□□□□□□□□□□□□□□□□□□□□□▪▪▪▪▪▪▪▪▪	Beijing	22333273733
3	MP283202	▪▪▪▪▪▪▪▪▪▪▪▪▪▪▪▪▪▪▪▪▪▪▪▪▪▪▪▪▪▪▪▪□□□□▪▪▪▪▪▪▪	T1	22436153321
4	MP391501	▪▪▪▪▪▪▪▪▪▪▪▪▪▪▪▪▪▪▪▪▪▪▪▪▪▪▪▪▪▪▪▪□□□□▪▪▪▪▪▪▪	T1	26536221531
5	MP26440	▪▪▪▪▪▪▪▪▪▪▪▪▪▪▪▪▪▪▪▪▪▪▪▪▪▪▪▪□□□□▪□▪▪□▪▪▪▪▪▪	EAI5	24536221533
6	MP272401	▪▪▪▪▪▪▪▪▪▪▪▪▪▪▪▪▪▪▪▪▪▪▪▪▪▪▪▪□□□□▪□▪▪□▪▪▪▪▪▪	EAI5	24536221431
7	MP327701	▪▪▪▪▪▪▪▪▪▪▪▪▪▪▪▪▪▪▪▪▪▪▪▪▪▪▪▪□□□□▪□▪▪□▪▪▪▪▪▪	EAI5	25433321232
8	MP29002	▪▪▪▪▪▪▪▪▪▪▪▪▪▪▪▪▪▪▪▪▪▪▪▪▪▪▪▪□□□□□□▪▪□▪▪▪▪▪▪	ND	24536221532
9	MP307404	▪▪▪▪▪▪▪▪▪▪▪▪▪▪▪▪▪▪▪▪▪▪▪▪▪▪▪▪□□□□□□▪▪□▪▪▪▪▪▪	ND	24536221532
10	MP322803	▪▪▪▪▪▪▪▪▪▪▪▪▪▪▪▪▪▪▪▪▪□□□□▪□□□□□□□□▪▪□▪▪▪▪▪▪	ND	24443321233
11	MP338904	▪▪▪▪▪▪▪▪▪▪▪▪▪▪▪▪▪▪▪▪▪□□□□▪□□□□□□□□▪▪□▪▪▪▪▪▪	ND	24443321233
12	MP329301	▪▪▪▪▪▪▪▪▪▪▪▪▪▪▪▪▪▪▪▪▪□□□□▪□□□□□□□□▪▪□▪▪▪▪▪▪	ND	22333253533
13	MP43301	▪▪▪▪▪▪▪▪▪▪▪▪▪▪▪▪▪▪▪▪▪▪▪▪▪▪▪▪▪▪▪▪□□▪▪▪▪▪▪▪▪▪	MANU2	22645173532
14	MP26204	▪▪▪□□▪▪▪▪▪▪▪▪▪▪▪▪▪▪▪▪▪▪▪▪▪▪▪▪▪▪▪□□▪▪▪▪▪▪▪▪▪	ND	22745173533
15	MP82703	▪▪▪□□□□□□□□□▪▪▪▪▪▪▪▪▪▪▪▪▪▪▪▪▪▪▪▪□□□□▪▪▪▪▪▪▪	T1	22535153324
16	MP79701	▪▪▪▪▪▪▪▪▪▪▪▪▪▪▪▪▪▪▪▪▪▪▪▪▪▪▪▪▪▪▪▪□▪▪□▪▪▪▪▪▪▪	ND	24433221533
17	MP48404	▪▪▪▪▪▪▪▪▪▪▪▪▪▪▪▪▪▪▪▪▪▪□▪▪▪▪▪□□□□▪□▪▪□▪▪▪▪▪▪	EAI6_BGD1	24536221731
18	MP252901	▪▪▪▪▪▪▪▪▪▪▪▪▪▪▪▪▪▪▪▪▪▪▪▪▪▪▪▪□□□□▪□▪▪▪▪▪□▪▪▪	EAI1_SOM	21535223512
19	MP245201	▪▪▪▪▪▪▪▪▪▪□▪▪▪▪▪▪▪▪▪▪▪▪▪▪▪▪▪□□□□□□▪▪▪▪▪□▪▪▪	ND	25536223512
20	MP338504	▪▪▪▪▪▪▪▪▪▪▪▪□▪▪▪▪▪▪▪▪▪▪▪▪▪▪▪□□□□▪□▪▪▪▪▪□□□□	ND	35531323213

## Discussion

There is lack of data on the prevalence of TB in urban slums of Bangladesh. According to the recently completed national TB prevalence survey in Bangladesh, the prevalence of new SS+ TB was estimated to be 79.4 per 100,000 and the prevalence rate for urban area was 51.1 per 100,000 [34]. Whereas our study, conducted in an urban slum area, revealed high prevalence of TB which is more than two times higher than overall prevalence and nearly four times higher than the prevalence in urban settings. This study has provided insight into the prevalence of TB, increased the case detection and identified some of the contributing factors for increased rate of SS+ PTB in a densely populated urban slum in Bangladesh.

This study also showed that active case finding for TB in urban slum setting is effective, given the high rate of participation, the feasibility of timely transportation of collected specimens to the central laboratory (no contamination), as well as laboratory testing which were used to investigate the TB burden along with molecular epidemiology of TB in settings like an urban slums.

The study results of our study indicate that clinical variables like cough <3 weeks and low BMI should be considered for suspecting TB cases and these issues should be addressed in the current NTP guidelines and awareness campaign which usually addresses only those with cough for at least three weeks. This could eventually detect those masked TB cases without cough or cough for less than three weeks duration. According to existing NTP guidelines in Bangladesh a patient is not suspected to have TB unless the patient has been coughing for three or more weeks. The gap of this current criteria poses the risk that we might be missing quite a large number of TB cases. Our study indicates that pulmonary TB with a history of cough is not significantly less frequent, when duration of cough is shorter than three weeks. This observation was also done in the prevalence study where the majority of TB cases identified did not have a history of cough [8]. One of the recent studies also presents the data with relevance of duration of cough and TB in line with our findings [35]. Several studies in the past showed an association between malnutrition and TB [36], [37], [38] and in one of our previous studies in prison we confirmed that in the prison population in Bangladesh [17]. The presence of low BMI in such a population is an important screening indicator of the disease in this population. However, more research with larger population should be performed to support these findings among different population group of Bangladesh.

In our study, among the detected cases the male-female ratio was 1.6∶1. In recently completed prevalence survey, TB in males was three times higher than females [34]. Among the adult population a higher male: female ratio has also been found in data from the NTP report [5]. However, we cannot comment whether TB is more common in men or we have missed the women with TB due to their perceptions of TB, denial about the suspicion of having TB related with social stigma or their ability to produce quality sputum specimen. It is evident that, women experienced longer delays in help seeking for TB at different stages of the disease ccompared with men [39]. There is definite need of study addressing gender differences in TB diagnosis and treatment to see the gender variations in treatment seeking behaviour in the community.

One of the objectives of our study was to characterize those strains that caused TB in an urban slum of Bangladesh and to investigate the extent of transmission. However, it is difficult to draw a conclusion from our study results as we have smaller numbers of detected cases and the study period was short to observe the transmission pattern. A relatively higher proportion (20%) of our *M. tuberculosis* isolates were clustered during this short study period compared to 11% cluster in our previous study in the rural community [10]. There was no apparent epidemiological link among the clustered cases. However, there is every possibility of recent transmission of TB among the infected persons considering the fact that this study was performed in an urban slum which was overcrowded and congested. Interestingly, the clustering was found among the younger age group (mean age 29; range: 19–40 yrs) which also favours recent transmission. It might not be possible to find the index case but considering the TB incubation period ranging from few months to few years there is possibility of recent transmission. It is important to determine whether TB disease has resulted from recent exogenous infection/reinfection or endogenous reactivation of a long-term latent infection to have an effective TB control measure strategy [40]. The results showed that it is feasible to conduct this transmission study in urban slum settings and being a high burden country, Bangladesh warrants these transmission dynamic studies in larger scale.

One of the limitations of our study was the purposive selection of Mirpur urban slum. This was selected as the area had been used as field sites for different studies conducted by icddr,b and we have a harmonious relationship with the population of the study area. We have not taken the HIV status into consideration as the prevalence of HIV in Bangladesh is low (less than 1%). Another reason for not taking the HIV status into consideration was the requirement of voluntary counseling prior asking the patient about the HIV status, which was difficult in active screening based field study. However, we believe that the interpretation of results in our study has not been influenced by the HIV status. It is important to conduct a prospective study with a larger sample size in the urban slum settings to estimate the prevalence of TB and its transmission in these high risk group. The current study identified areas in which design and data collection can be strengthened. It will be interesting to understand and know how the programmatic factors like screening only for chronic cough (less sensitivity), diagnosis by sputum smear microscopy (<70% sensitivity), others (services, human resource, quality etc.) and non-programmatic factors like care seeking, private sector, socioeconomic status etc. affecting detection of SS+ TB cases in urban improvised areas. This system bypass or non use of or non-detection by DOTS is important and should be addressed particularly with the growing threat of HIV infection and drug resistant TB in the country.

## References

[1] WHO (2011) Global tuberculosis control: WHO report 2011: World Health Organization.

[2] Hayward A, Darton T, Van-Tam J, Watson J, Coker R, et al (2003) Epidemiology and control of tuberculosis in Western European cities. The International Journal of Tuberculosis and Lung Disease 7: 751–757. 12921151

[3] Braden CR, Templeton GL, Cave MD, Valway S, Onorato IM, et al (1997) Interpretation of restriction fragment length polymorphism analysis of Mycobacterium tuberculosis isolates from a state with a large rural population. Journal of Infectious Diseases 175: 1446. 9180185 10.1086/516478

[4] Hossain S, Quaiyum MA, Zaman K, Banu S, Husain MA, et al (2012) Socio Economic Position in TB Prevalence and Access to Services: Results from a Population Prevalence Survey and a Facility-Based Survey in Bangladesh. PloS one 7: e44980. 23028718 10.1371/journal.pone.0044980 PMC3459948

[5] Hossain S, Quaiyum MA, Zaman K, Banu S, Husain MA, et al (2012) Socio Economic Position in TB Prevalence and Access to Services: Results from a Population Prevalence Survey and a Facility-Based Survey in Bangladesh. PLoS One 7: e44980. 23028718 10.1371/journal.pone.0044980 PMC3459948

[6] Ford CM, Bayer AM, Gilman RH, Onifade D, Acosta C, et al (2009) Factors associated with delayed tuberculosis test-seeking behavior in the Peruvian Amazon. The American journal of tropical medicine and hygiene 81: 1097–1102. 19996443 10.4269/ajtmh.2009.08-0627 PMC2912503

[7] Asch S, Leake B, Anderson R, Gelberg L (1998) Why do symptomatic patients delay obtaining care for tuberculosis? American journal of respiratory and critical care medicine 157: 1244–1248. 9563746 10.1164/ajrccm.157.4.9709071

[8] Zaman K, Hossain S, Banu S, Quaiyum M, Barua P, et al (2012) Prevalence of smear-positive tuberculosis in persons aged 15 years in Bangladesh: results from a national survey, 2007-2009. Epidemiology and Infection 140: 1018–1027. 21880168 10.1017/S0950268811001609

[9] (DGHS) DGoHS (2011) ‘Tuberculosis Control in Bangladesh’ Annual Report 2011. National Tuberculosis Control Programme (NTP).

[10] Banu S, Uddin M, Islam M, Zaman K, Ahmed T, et al (2012) Molecular epidemiology of tuberculosis in rural Matlab, Bangladesh. The International Journal of Tuberculosis and Lung Disease 16: 319–326. 22640444 10.5588/ijtld.11.0426

[11] Van Embden J, Cave M, Crawford J, Dale J, Eisenach K, et al (1993) Strain identification of Mycobacterium tuberculosis by DNA fingerprinting: recommendations for a standardized methodology. Journal of Clinical Microbiology 31: 406–409. 8381814 10.1128/jcm.31.2.406-409.1993 PMC262774

[12] Supply P, Magdalena J, Himpens S, Locht C (1997) Identification of novel intergenic repetitive units in a mycobacterial two-component system operon. Molecular microbiology 26: 991–1003. 9426136 10.1046/j.1365-2958.1997.6361999.x

[13] Supply P, Mazars E, Lesjean S, Vincent V, Gicquel B, et al (2000) Variable human minisatellite-like regions in the Mycobacterium tuberculosis genome. Molecular microbiology 36: 762–771. 10844663 10.1046/j.1365-2958.2000.01905.x

[14] Hawkey PM, Smith EG, Evans JT, Monk P, Bryan G, et al (2003) Mycobacterial interspersed repetitive unit typing of Mycobacterium tuberculosis compared to IS6110-based restriction fragment length polymorphism analysis for investigation of apparently clustered cases of tuberculosis. Journal of Clinical Microbiology 41: 3514–3520. 12904348 10.1128/JCM.41.8.3514-3520.2003 PMC179797

[15] Mazars E, Lesjean S, Banuls A-L, Gilbert M, Vincent V, et al (2001) High-resolution minisatellite-based typing as a portable approach to global analysis of Mycobacterium tuberculosis molecular epidemiology. Proceedings of the National Academy of Sciences 98: 1901–1906. 10.1073/pnas.98.4.1901 PMC29354 11172048

[16] Tessema B, Beer J, Merker M, Emmrich F, Sack U, et al (2013) Molecular epidemiology and transmission dynamics of Mycobacterium tuberculosis in Northwest Ethiopia: new phylogenetic lineages found in Northwest Ethiopia. BMC infectious diseases 13: 1–11. 23496968 10.1186/1471-2334-13-131 PMC3605317

[17] Banu S, Hossain A, Uddin MKM, Uddin MR, Ahmed T, et al (2010) Pulmonary tuberculosis and drug resistance in Dhaka central jail, the largest prison in Bangladesh. PloS one 5: e10759. 20505826 10.1371/journal.pone.0010759 PMC2874010

[18] Graham SM, Ahmed T, Amanullah F, Browning R, Cardenas V, et al (2012) Evaluation of tuberculosis diagnostics in children: 1. Proposed clinical case definitions for classification of intrathoracic tuberculosis disease. Consensus from an expert panel. Journal of Infectious Diseases 205: S199–S208. 22448023 10.1093/infdis/jis008 PMC3334506

[19] Stegen G, Jones K, Kaplan P (1969) Criteria for guidance in the diagnosis of tuberculosis. Pediatrics 43: 260–263. 5304285

[20] Mehnaz A, Arif F (2005) Applicability of scoring chart in the early detection of tuberculosis in children. Journal of the College of Physicians and Surgeons—Pakistan: JCPSP 15: 543. 16181573

[21] Petroff S (1915) A new and rapid method for the isolation and culture of tubercle bacilli directly from the sputum and faeces. J Exp Med 21: 38–42. 19867850 10.1084/jem.21.1.38 PMC2125265

[22] Banu S, Mahmud AM, Rahman MT, Hossain A, Uddin MKM, et al (2012) Multidrug-Resistant Tuberculosis in Admitted Patients at a Tertiary Referral Hospital of Bangladesh. PloS one 7: e40545. 22808189 10.1371/journal.pone.0040545 PMC3394739

[23] Canetti G, Fox W, Khomenko Aa, Mahler H, Menon N, et al (1969) Advances in techniques of testing mycobacterial drug sensitivity, and the use of sensitivity tests in tuberculosis control programmes. Bulletin of the World Health Organization 41: 21. 5309084 PMC2427409

[24] Kamerbeek J, Schouls L, Kolk A, van Agterveld M, van Soolingen D, et al (1997) Simultaneous detection and strain differentiation of Mycobacterium tuberculosis for diagnosis and epidemiology. J Clin Microbiol 35: 907–914. 9157152 10.1128/jcm.35.4.907-914.1997 PMC229700

[25] Kox LF, Rhienthong D, Miranda AM, Udomsantisuk N, Ellis K, et al (1994) A more reliable PCR for detection of Mycobacterium tuberculosis in clinical samples. J Clin Microbiol 32: 672–678. 8195377 10.1128/jcm.32.3.672-678.1994 PMC263105

[26] Van Soolingen D, De Haas P, Hermans P, Groenen P, Van Embden J (1993) Comparison of various repetitive DNA elements as genetic markers for strain differentiation and epidemiology of Mycobacterium tuberculosis. Journal of clinical microbiology 31: 1987. 7690367 10.1128/jcm.31.8.1987-1995.1993 PMC265684

[27] Brosch R, Gordon SV, Marmiesse M, Brodin P, Buchrieser C, et al (2002) A new evolutionary scenario for the Mycobacterium tuberculosis complex. Proc Natl Acad Sci U S A 99: 3684–3689. 11891304 10.1073/pnas.052548299 PMC122584

[28] Mazars E, Lesjean S, Banuls AL, Gilbert M, Vincent V, et al (2001) High-resolution minisatellite-based typing as a portable approach to global analysis of Mycobacterium tuberculosis molecular epidemiology. Proceedings of the national academy of Sciences 98: 1901. 10.1073/pnas.98.4.1901 PMC29354 11172048

[29] Banu S, Gordon SV, Palmer S, Islam R, Ahmed S, et al (2004) Genotypic analysis of Mycobacterium tuberculosis in Bangladesh and prevalence of the Beijing strain. Journal of clinical microbiology 42: 674–682. 14766836 10.1128/JCM.42.2.674-682.2004 PMC344461

[30] (2009) National Guidelines and Operational Manual for Tuberculosis Control: National Tuberculosis Control Programme, Bangladesh.

[31] Sola C, Filliol I, Legrand E, Mokrousov I, Rastogi N (2001) Mycobacterium tuberculosis phylogeny reconstruction based on combined numerical analysis with IS 1081, IS 6110, VNTR, and DR-based spoligotyping suggests the existence of two new phylogeographical clades. Journal of molecular evolution 53: 680–689. 11677628 10.1007/s002390010255

[32] Brosch R, Gordon SV, Marmiesse M, Brodin P, Buchrieser C, et al (2002) A new evolutionary scenario for the Mycobacterium tuberculosis complex. Proceedings of the national academy of Sciences 99: 3684. 10.1073/pnas.052548299 PMC122584 11891304

[33] Gagneux S, Small PM (2007) Global phylogeography of Mycobacterium tuberculosis and implications for tuberculosis product development. The Lancet infectious diseases 7: 328–337. 17448936 10.1016/S1473-3099(07)70108-1

[34] Zaman K, Hossain S, Banu S, Quaiyum M, Barua P, et al (2011) Prevalence of smear-positive tuberculosis in persons aged⩾ 15 years in Bangladesh: results from a national survey, 2007–2009. Epidemiology and Infection 1: 1–10. 10.1017/S0950268811001609 21880168

[35] Ngadaya ES, Mfinanga GS, Wandwalo ER, Morkve O (2009) Detection of pulmonary tuberculosis among patients with cough attending outpatient departments in Dar Es Salaam, Tanzania: does duration of cough matter? BMC health services research 9: 112. 19570233 10.1186/1472-6963-9-112 PMC2713219

[36] Karyadi E, Schultink W, Nelwan RHH, Gross R, Amin Z, et al (2000) Poor micronutrient status of active pulmonary tuberculosis patients in Indonesia. The Journal of nutrition 130: 2953–2958. 11110853 10.1093/jn/130.12.2953

[37] Onwubalili J (1988) Malnutrition among tuberculosis patients in Harrow, England. European journal of clinical nutrition 42: 363. 3396528

[38] Saha K, Rao K (1989) Undernutrition in lepromatous leprosy. V. Severe nutritional deficit in lepromatous patients co-infected with pulmonary tuberculosis. European journal of clinical nutrition 43: 117. 2707215

[39] Karim F, Islam MA, Chowdhury A, Johansson E, Diwan VK (2007) Gender differences in delays in diagnosis and treatment of tuberculosis. Health policy and planning 22: 329–334. 17698889 10.1093/heapol/czm026

[40] Cohen T, Colijn C, Finklea B, Murray M (2007) Exogenous re-infection and the dynamics of tuberculosis epidemics: local effects in a network model of transmission. J R Soc Interface 4: 523–531. 17251134 10.1098/rsif.2006.0193 PMC2373405

